# Molecular Detection and Conventional Identification of *Leishmania* Species in Reservoir Hosts of Zoonotic Cutaneous Leishmaniasis in Fars Province, South of Iran

**Published:** 2013

**Authors:** A Mirzaei, S Rouhani, PA Kazerooni, M Farahmand, P Parvizi

**Affiliations:** 1 Molecular Systematic Laboratory, Parasitology Department, Pasteur Institute of Iran, Tehran, Iran; 2 Medical Faculty, Ilam University of Medical Sciences, Ilam, Iran; 3 Parasitology Department, Medical Faculty, Shahid Beheshti University of Medical Sciences, Iran; 4 Shiraz University of Medical Sciences, Shiraz, Iran

**Keywords:** *Leishmania major*, ITS-ribosomal DNA, Nested PCRs, Rodents, Iran

## Abstract

**Background:**

The objectives of our research were to search for *Leishmania* species in rodents in Fars province, south of Iran, and to compare molecular with conventional methods for detecting these parasites.

**Methods:**

Rodents were captured using live traps and screened for *Leishmania* species using molecular and conventional methods, including the taking of smears from each ear. Nested PCR was employed to detect *Leishmania* in rodents by amplifying a region of the ribosomal RNA amplicon of *Leishmania* (ITS1-*5.8S rRNA*-ITS2) that is species-specific by DNA sequence.

**Results:**

Totally, 122 rodents were captured. *Leishmania* parasites were detected using the nested PCR and three conventional methods (direct smear, NNN culture and Balb/C inoculation. 41 (33.6%) out of 122 rodents had *Leishmania* infections (34 Meriones lybicus and 7 M. persicus). All PCR products of the ITS-rDNA gene were sequenced. Sequence analysis revealed that 28 out of 41 positive samples were *Leishmania major*. Thirteen sequences were unreadable and therefore not identified.

**Conclusion:**

At least two gerbil species common in Fars ZCL foci, M. lybicus and M. persicus, are acquiring infections of L. major and may be reservoir hosts of one predominant parasite haplotype. Most infections were detected molecularly not by conventional methods, because most rodents died in the traps.

## Introduction

In Fars province, south of Iran, Zoonotic Cutaneous Leishmaniasis (ZCL) is geographically widely distributed in many districts, locations, and villages. It is a parasitic disease caused by infection with protozoan parasites of *Leishmania major* (Kinetoplastidae: Trypanosomatidae), and ZCL has increased in the past five years in Fars Province (1). *Leishmania major* is believed to predominate in or near the burrows of rodents, where it is transmitted by the bites of phlebotomine sandflies (Diptera: Psychodidae) of the genus *Phlebotomus* (1–4).

Currently worldwide, at least 22 species of *Leishmania* are pathogenic for humans, who are infected when exposed to a natural transmission cycle (5–6). Wild and domesticated mammals act as reservoirs of zoonotic infections and humans can be sources of anthroponotic infections (7).

ZCL caused by *L. major* has been recorded in some locations of southern Iran (8, 9). Recently, some regions in Fars Province have become focal points for research on endemic ZCL due to the significant increase in the number of ZCL cases over the past few years (4). The causative organisms of ZCL in Iran have been characterized on the basis of clinical symptoms, geographical locality or specific reservoir hosts, infection of experimental animals, species-specific monoclonal antibodies, microscopic observation, parasite cultivation and molecular methods (4, 10–16).

Recently, molecular markers have increasingly been used for epidemiological studies on leishmaniasis, in order to identify and type *Leishmania* at the genus, species or strain level. Good molecular tools should be easy to use, rapid, and show high sensitivity and specificity in order to identify accurately parasites in vectors and reservoir hosts. Several molecular methods have been introduced for identification and characterization of *Leishmania* (17, 18).

Culture of promastigotes from infected tissues and direct identification of amastigotes in microscope smears have long been considered the standard for diagnosis in Iran. While these techniques are highly specific for diagnosing leishmaniasis, they are not always sensitive. The different species of *Leishmania* are not equally easy to culture; contamination is a constant hazard, and the percent success for microscopic identification of amastigotes in stained preparations varies depending on the number of parasites present and/or the experience of the person examining the slide. Unfortunately, today there is no single widely accepted standard procedure that can be used as a basis for evaluating new molecular diagnostic assays for leishmaniasis (4, 10, 17).

Iranian investigators have reported rodent reservoirs of ZCL with different regional importance: the gerbils *Rhombomys opimus* and *Meriones libycus* in the northeast and centre; the rodents *Meriones libycus*, *Tatera indica* and *Nesokia indica* in the centre and southwest; the gerbil *Meriones hurrianae* in the southeast; and the gerbil *Tatera indica* in the southwest and south. Most of the infections were not typed (15, 19, 20). For the current investigation, nested polymerase chain reaction (PCR) was used by targeting a fragment of the ITS-ribosomal RNA (rDNA) region, consisting of ITS1-5.8S–rRNA-ITS2 gene, following the strategy of Parvizi et al. (2005) and Cupolillo et al. (1995) (21, 22).

The first objective of our current research was to search for *Leishmania* parasites in rodents in Fars Province. However, although there are records of *Leishmania* in some species of rodents including *M. libycus* in Fars, the parasites were not identified molecularly. Our second objective was to compare molecular with conventional methods for detecting *Leishmania* in Iranian rodents.

## Materials and Methods

### Rodent collections within the ZCL focus

**Fig. 1:**
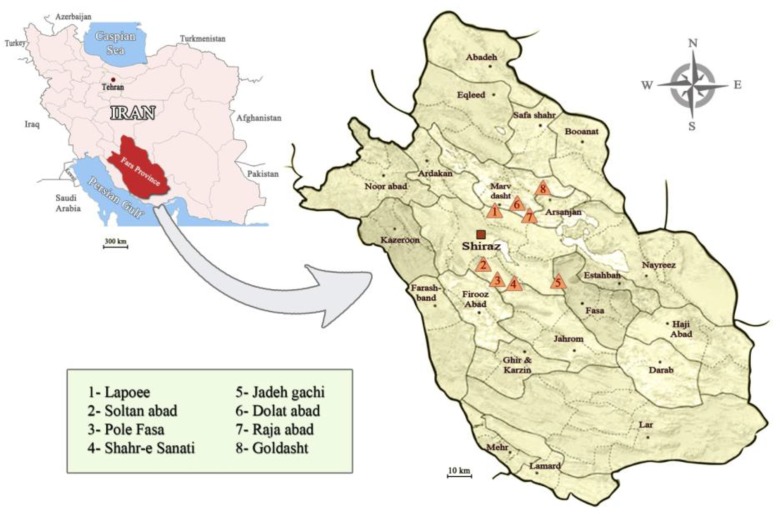
Location of villages and regions, Fars Province of Iran, where rodents were sampled

The investigation was carried out in 8 villages within an important ZCL focus in Fars Province, south of Iran (Fig. 1). The choice of these locations was based on the increased number of reported cases of ZCL by Fars Health Centre. The disease focus is at an altitude of 1400-1800 metres above sea level. Fars Province is known to be a suitable habitat for the proliferation of the vector, reservoir and the parasite, and it has unique geographical, ecological and climatic conditions (23).

After identifying active colonies of rodents (Fig. 1, Tables 1, 2), the collections were carried out in 2009 and 2010, in 8 villages in three districts (Marvdasht, Ghareh Bagh and Zarghan) of Fars Province.

**Table 1:** Distribution of rodents in different villages based on dates of collections in our ZCL sites study in Fars Province

	2008																					2009																						Total
Date	2008/Aug/14			2008/Aug/15		2008/Aug/16		2008/Aug/17	2008/Aug/18		2008/Aug/19		2008/Aug/20	2008/Aug/21		2008/Nov/5		2008/Nov/8	2008/Nov/11	2008/Nov/12		2009/Aug/4	2009/Aug/5	2009/Aug/6	2009/Aug/7	2009/Aug/8		2009/Aug/9	2009/Aug/10	2009/Aug/11	2009/Aug/12	2009/Oct/19	2009/Oct/20		2009/Oct/21		2009/Oct/22		2009/Oct/23	2009/Oct/24		2009/Oct/25		
Villages Rodent Spp.	soltanabad	shahraksanati	polefasa	polefasa	soltanabad	Jadegachi	polefasa	Jadegachi	Jadegachi	shahraksanati	Dolatabad	Goldasht	Goldasht	Goldasht	Rajaabad	polefasa	Jadegachi	Jadegachi	Jadegachi	polefasa	Jadegachi	soltanabad	soltanabad	Lapoee	Lapoee	Lapoee	Dolatabad	Dolatabad	Dolatabad	Dolatabad	Dolatabad	Lapoee	Lapoee	Dolatabad	Dolatabad	Lapoee	Lapoee	Dolatabad	Lapoee	Lapoee	Dolatabad	shahraksanati	Lapoee	
***M. libycus***	2	1	2	3	1	2	1	1	0	1	10	4	4	9	1	1	0	0	2	1	1	0	1	1	2	5	1	1	3	2	3	8	5	2	1	7	1	1	4	2	1	2	2	102
***M. persicus***		2							2		1						1	1	4	2										2												4		19
***Rattus rattus***																						1																						1
**Total**	2	3	2	3	1	2	1	1	2	1	11	4	4	9	1	1	1	1	6	3	1	1	1	1	2	5	1	1	3	4	3	8	5	2	1	7	1	1	4	2	1	6	2	122
**percents**	1.64%	2.46%	1.64%	2.46%	0.82%	1.64%	0.82%	0.82%	1.64%	0.82%	9%	3.4%	3.4%	7.4%	0.82%	0.82%	0.82%	0.82%	4.72%	2.46%	0.82%	0.82%	0.82%	0.82%	1.64%	4.1%	0.82%	0.82%	2.46%	3.4%	2.46%	6.56%	4.1%	1.64%	0.82%	5.74%	0.82%	0.82%	3.4%	1.64%	0.82%	4.92%	1.64%	100%

**Table 2:** Prevalence of rodent-caught populations and their *Leishmania* ITS-rDNA gene infections in alive and dead captured rodents in different locations, in Fars Province

Capture year	Sample No.	Alive captured					Sample No.	Dead captured					Total	
		*Leishmania* +ve						*Leishmania* +ve						
		Species	Right ear	Left ear	Both ears	Total +ve		Species	Right ear	Left ear	Both ears	Total +ve	*Leishmania* +ve	Samples
2008	0	*L. major*	0	0	0	0	60	*L. major*	11	3	3	17	17	60
		unidentified	0	0	0	0		unidentified	4	2		6	6	
		*L. major* & unidentified	0	0	0	0		*L. major* & unidentified	0	0	0	0	0	
2009	14	*L. major*	3	2	0	5	48	*L. majo*r	2	4	1	7	12	62
		Unidentified	2	0	0	2		unidentified	1	1		2	4	
		*L. major* & unidentified	0	0	0	0		*L. major* & unidentified	0	0	2	2	2	
Total	14	-	5	2	0	7	108	-	18	10	6	34	41	122

**Table 3:** Results of conventional and molecular methods for screening *Leishmania* parasite in captured rodent species in our ZCL sites study in Fars Province

Methods		Conventional methods						Molecular methods			Parasite Spp.	
		Balb/C inoculation		NNN medium		Direct slide		ITS-rDNA				
Capture year↓	Rodent species→	*M.lybicus*	*M.persicus*	*M.lybicus*	*M.persicus*	*M.lybicus*	*M.persicus*	*M.lybicus*	*M.persicus*	Total	*L.major*	Unidentified
2009	Result	+Ve/Total	+Ve/Total	+Ve/Total	+Ve/Total	+Ve/Total	+Ve/Total	+Ve/Total	+Ve/Total	+Ve/Total		
	August	0	0	0	0	0	0	18/42	4/5	22/47	16	6
	November	0	0	0	0	0	0	1/5	2/8	3/13	2	1
2010	August	1/2	0	0/2	0	2/2	0	2/19	0/2	2/21	1	1
	October	4/11	0	2/11	0	4/11	0	13/36	1/4	14/40	9	5
Total		5/13	0	2/13	0	6/13	0	34/102	7/19	41/121	28	13

The villages sampled for the current investigation were: 4 villages from Ghareh Bagh district (1486 m a.s.l.; 52° 32 'N, 29° 36'E), 3 villages from Marvdasht district (1595 m a.s.l.; 52° 48'N, 29° 52'E), and 1 village from Zarghan District (1600 m a.s.l.; 52° 32 'N, 29° 36'E), all within the known focus of ZCL reported by the regional public health services for interventions. Rodent samples were collected from colonies located about 1 to 2 km around the villages. Each visit, 40–50 live traps were used and the traps were baited with cucumber and placed in active burrows. The traps were set up early in the morning and evening in August and November 2009 and also August and October in 2010. The trapped rodents were transferred to the animal house facility in the Pasteur Institute of Iran, Tehran, and maintained for parasitological and molecular testing. The morphological identifications of specimens were made using external criteria of color, body measurements, ears, tail, feet, teeth and cranium for each specimen and also by their species-specific Cyt b sequences. (21, 24, 25). In the laboratory, the captured rodents were examined for any lesions while under ether anesthesia. After crushing ear skin, smears were taken for light microscopy and serous fluid inoculated into NNN medium. Two impression smears were prepared from the ears of each rodent, fixed in methanol and stained by the Giemsa method, before being examined carefully under the light microscope for amastigote forms. NNN cultures were incubated at 25°C for up to 4 weeks with weekly subcultures. First appearance and the growth of promastigotes were regularly monitored. Any contaminated culture was destroyed. Samples with high numbers of organism colonies were transferred to RPMI medium containing 15% fetal calf serum (Gibco).

Samples from the ears of each rodent were injected subcutaneously at the base of the tail of Balb/C mice (1 per ear). A weekly observation for any cutaneous lesions was performed on inoculated Balb/C for 6 months. A direct smear was taken from the infected Balb/c to confirm the presence of amastigotes. DNA extraction and screening for parasites by PCR methods were done afterwards. The records of all the results were kept. The polymerase chain reaction (PCR) method as a species identification procedure is described in the following text.

### DNA extraction and nested PCR of ITS-rDNA gene of Leishmania species for amplifying ITS1-5.8S rRNA -ITS2

DNA extraction from each ear of rodents and any parasites within was done using the previous studies (3, 15). All rodents samples were screened for *Leishmania* parasites by nested PCR of a fragment of ITS -rDNA. Forward primer IR1 (5' GCTGTAGGTGAACCTGCAGCAGCTGGATCATT 3') was used with the reverse primer IR2 (5' GCGGGTAGTCC TGCCAAACACTCAGGTCTG 3') for the first stage; and then forward primer ITS1F (5' GCAGCTGGATCATTTTCC 3') and reverse primer ITS2R4 (5' ATATGCAGAAGAGAGGAGGC 3') were used to amplify the ITS1-5.8S- ITS2 fragment for the second stage of the nested PCR (2). For PCR amplification, DNA of known *L. major* was used as a positive control and PCR water as a negative control. PCR products were directly sequenced to identify *Leishmania* species, strains and haplotypes infecting individual rodent, and all haplotypes were identified to species by phylogenetic analysis. To do this, DNA sequences were edited and aligned using Sequencher 4.10 software for PC, and the multiple alignments of new DNA haplotypes and homologous GenBank sequences were exported into MEGA software for phylogenetic analysis.

## Results

### Collections of rodents and use of conventional methods to detect Leishmania in them

In this study, 122 rodents were captured (Fig. 1, Table 1). Among these, 102 were identified morphologically as *M. lybicus*, 19 as *M. persicus* and one as *Rattus rattus*. Fourteen out of 122 rodents were captured alive and sampled to isolate and detect *Leishmania* using both conventional and molecular methods. Totally, 108 out of 122 rodents were captured dead and sampled to detect *Leishmania* using only molecular methods (Table 2).

Seven out of 14 (42.85%) live-caught rodents were *Leishmania* positive. Of these, 6 were positive using direct smear and microscopic observation, and 5 by inoculation in Balb/C mice that produced ulcers on the base of the tail (Table 2).

Five out of the 14 rodents (35.71%) were *Leishmania* positive by both conventional and molecular methods. In total, 6 (42.85%) of these rodents were *Leishmania* positive using at least one conventional method. Only 2 of the samples grew in NNN medium without contamination.

### Leishmania infections in rodents identified by Nested PCR of ITS1-rDNA-ITS2

For identifying species of *Leishmania*, both ears of all 122 collected rodents were screened by amplifying two fragments of ITS-rDNA by nested PCR. 41 out of 122 (33.6%) rodents were *Leishmania* positive (34 out of 102 *M. lybicus* and 7 out of 19 *M. persicus*). Only 6 of the infected rodents had *Leishmania* infections in both ears and only 6 were positive by conventional methods too (Table 2).

**Fig. 2:**
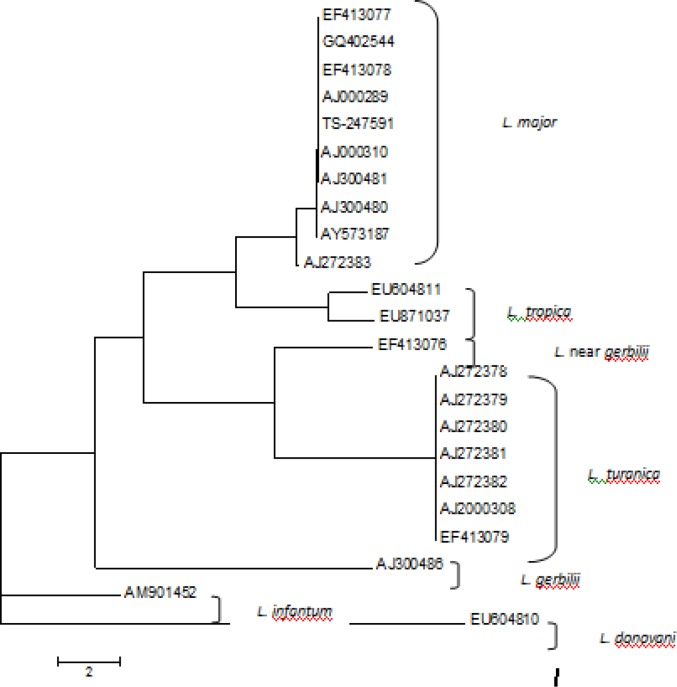
Unrooted neighbor-joining tree showing the relationships of the haplotypes of the ITS1-5.8SrRNA gene fragment for the isolates of *Leishmania* species in rodents in Fars Province and in GenBank

All positive PCR products of ITS-rDNA in rodents were sequenced. Sequence analysis revealed 28 out of 41 positive samples were *L. major*. 13 sequences had unreadable sequences that could not be identified. Among the 28 *L. major* infections of rodent species with different origins, 26 were of a single haplotype, sharing 100% sequence identity with GenBank sequences of *L. major* from other regions of Iran and elsewhere (Fig. 2). Two specimens (*M. libycus)* had a novel haplotype, but this differed by only one nucleotide from the common haplotype of *L. major*.

## Discussion

Two haplotypes of ITS-rDNA gene of *L. major* were identified by aligning sequences and comparing them with those submitted in GenBank. They were discovered in two gerbil species, *M. lybicu*s and *M. persicus*, in Fars province. A common haplotype was identical to two GenBank sequences from strains of *L. major* originating from Sudan and Iran (GenBank accessions EF413075; AJ300481) (Table 2). Most *M. lybicu*s and *M. persicus* were infected with this common haplotype (26/28 infections with readable sequences). The second haplotype of *L. major* was found to be novel (GenBank accession number KF152937).).

Totally, 23 haplotype sequences of the ITS-rDNA were aligned for phylogenetic analysis in MEGA. These sequences included all the strains of *Leishmania* found in rodents captured in Fars province and those in GenBank known from the region. The phylogram made by the NJ method is presented in Fig. 2. Based on the ITS-rDNA fragment examined, most *Leishmania* species grouped in two main branches. The first main branch contained agents of cutaneous leishmaniasis and mammalian infections, and the second branch contained agents of visceral leishmaniasis. The first branch had species-specific subgroups.

Thirteen rodents were positive for *Leishmania* infections but the sequences were unreadable, and so *Leishmania* species were not identified. These unreadable sequences may have come from mixed infections of two or more strains of *L. major* or of different *Leishmania* species (*L. major*, *L. turanica*, *L. gerbilli sensu lato* and *Leishmania* close to *L. gerbilli* or a related species) (2, 4, 26). Mixed infections of *Leishmania* species may complicate the detection of changes in the infection rates of *L. major* in different rodent species. *Rhombomys opimus* had mixed infections in the ex-U.S.S.R. (26). Mixed infections in rodents will not always be detected, because only the target DNA of the more abundant parasite will often be amplified by PCR. Using species-specific primers or cloning could help to sort out this point (2).

Many rodents from Iranian foci of rural ZCL have been found to be infected with *Leishmania* parasites (4, 15, 20, 27, 28). In some southern parts of Iran, *L. major* was isolated from *M. libycus* (16, 29, 30). *L. major* was also found in *M. libycus* in other regions, in Isfahan and Natanz in the centre of Iran, Damghan, Semnan Province, in Turkemen Sahara, Golastan Province (15, 19, 31, 32).

Various *Leishmania* parasites may infect *M. persicus* in Iran, with *L. major*, *L, donovani* and *L. infantum* being reported, for example from Meshkin-Shahr District in north west Iran, where visceral leishmaniasis is endemic (33, 34). Until now, there has been no report of finding *L. turanica* in *M. lybicu*s or *M. persicus*. In Iran, this parasite has been isolated and typed mostly from *R. opimus* (4, 19, 20). It is important to understand the distributions of all rodent species infected by *Leishmania*, and to identify which reservoirs are frequently infecting potential sandfly vectors (2, 26). Only *L. major* was detected in sandflies from the region of our rodent collections (4).

Controlling the rodent reservoir hosts of ZCL is a challenge to the health authorities in the southern parts of Iran. Changing social and economic conditions in the region may help increase the number of wild rodents and sand flies, and factors to consider include fast urbanization, new buildings on farms near rodent colonies, waste material storage, new agricultural projects, and the siting of animal shelters among old mud houses. These factors could facilitate the transmission of ZCL. Migrations of refugees from Afghanistan and neighboring countries might also help spread the disease (3, 30). Many researchers prefer to use either conventional or molecular methods to identify *Leishmania*, but in this report we employed both types of methods. It is important to underline that molecular methods are more efficient than conventional methods for screening rodent hosts, because conventional methods require the capture and maintenance of live animals. For such reasons, many investigators now use only molecular tools. However, conventional techniques can be appropriate in some circumstances, and so the efficiency of the two approaches should be compared.

## Conclusion

Our molecular method was more sensitive than the conventional methods, and it permitted the incrimination of two gerbil species, *M. lybicu*s and *M. persicus*, as regional reservoir hosts.
